# Economic recession and mental health distress among Japanese people in middle age

**DOI:** 10.1038/s41598-025-85198-6

**Published:** 2025-04-16

**Authors:** Hiroshi Murayama, Yukitsugu Komazawa, Masako Kakizaki, Yoshiharu Fukuda, Takahiro Tabuchi

**Affiliations:** 1 Research Team for Social Participation and Healthy Aging, Tokyo Metropolitan Institute for Geriatrics and Gerontology, 35-2 Sakae-cho, Itabashi-ku, Tokyo, 173-0015 Japan; 2 Almec Corporation, Tokyo, Japan; 3 Graduate School of Medical Sciences, Nagoya City University, Aichi, Japan; 4 Teikyo University Graduate School of Public Health, Tokyo, Japan; 5 Graduate School of Medicine, Tohoku University, Miyagi, Japan

**Keywords:** Economic recession, Mental health, Socioeconomic status, Middle age, Japan, Environmental social sciences, Health care

## Abstract

This study examined the association between the 2008 economic recession and mental health distress among middle-aged Japanese people, and whether sex and socioeconomic status affected this association. Data were obtained from a nationwide longitudinal study (“Longitudinal Survey of Middle-aged and Elderly Persons”), conducted since 2005 on randomly selected Japanese adults aged 50–59 years. Of the 34,240 respondents to the 2005 baseline survey, data for 33,815 who responded at least once both before 2007 and after 2008 were analyzed. The 6-item Kessler Psychological Distress Scale (K6) was used to assess mental health distress (i.e., K6 scores of ≥ 5). A generalized estimating equation model applied to 6 years of longitudinal data (2005–2010) showed that mental health worsened among men and women after the 2008 recession (odds ratio [95% confidence interval] = 1.11 [1.07–1.15] for men; 1.14 [1.10–1.18] for women), particularly among self-employed men (1.08 [1.02–1.14]). The mental health of women who graduated from junior high school worsened less than that of women who graduated from university or graduate school (0.89 [0.80–0.98]). This study confirmed the association between the 2008 economic recession and mental health distress and identified vulnerable socioeconomic groups. These findings provide useful information for future economic crises.

## Introduction

Economic adversity is one of the most important causes of mental health disorders and suicide^1–7^. There are strong associations between economic recessions and unemployment, income decline, home repossession, and unmanageable debts^8–12^. These factors substantially affect mental health, including the severity of stress, depression, and anxiety^8,9,11,13,14^. Higher stress levels increase national suicide rates, and the prevalence of psychiatric, alcohol-use, and substance-use disorders^11^. Among the various economic recessions in history, the 2007–2008 financial crisis that led to the so-called Great Recession affected several countries substantially. For instance, in contrast to the upward trend after 2002, Japan’s economy shrunk between 2008 and 2009. Japan’s gross domestic product (GDP) in 2008 and 2009 was  − 1.0% and  − 5.5%, respectively; from late 2008, the number of bankruptcies in construction, real estate, and production industries increased, and financial aid to small and medium enterprises decreased ^15^. The Great Recession of 2008 caused the European economy to shrink by 4%, and unemployment increased to 10% in 2009 ^3^.

Previous studies have examined the negative effects of the 2008 recession on mental health in European countries ^10,16,17^. Suicide rates increased considerably in the United Kingdom and Greece, as well as in the United States during this period ^18^. Financial hardship is a major outcome of economic recession, and can have negative effects on mental health ^8,13,14,19^. Countries such as Portugal reduced public spending in response to the economic crisis, which likely impacted the mental health of the population ^5^. Compared with countries that reduced spending on employment and social welfare, those with active policies to sustain welfare have lower suicide rates during economic recessions ^9^.

Vulnerable populations may be particularly exposed to the harmful effects of economic crises on mental health. A systematic literature review showed that the effect of economic recessions on individuals is modified by socioeconomic status (SES) ^8^. Research shows that three SES indicators—education, employment, and income—are each independently correlated with mental health status during economic recessions. For example, an Italian study found that in the Great Recession of 2008, people with higher levels of education tended to be at a greater risk of experiencing mental health problems ^13^. In Hungary, those unemployed during the Great Recession frequently experienced anxiety, depression, and low self-esteem^14^. A Greek study found that unemployed and unskilled workers were the most vulnerable in terms of psychological health during this period^20^. Regarding income, a Japanese study identified greater deterioration in the self-reported health of lower-income people compared to people with middle to higher incomes during the economic crisis^21^.

Sex may be an important demographic factor that affects mental health during recessions. Sex disparities have been frequently identified in mental health status before and after the 2008 Great Recession, particularly in the association between mental health problems and financial hardship. For instance, a study on the young adult labor force in Italy showed that men who were unemployed or insecurely employment during the 2008 Great Recession more likely to have experienced mental health issues^13^. Studies from Iceland and Sweden found that women experienced mental health deterioration during the recession, whereas a study in Spain found the same effect in men^22–24^.

While several studies have assessed the association between economic recessions and mental health disorders or distress, and examined the influences of sex and SES on this association, the results tend to vary by country^8^. Moreover, few studies have been conducted in non-Western countries such as Japan. Therefore, this study analyzed a nationally representative dataset of middle-aged adults in Japan to fulfill two specific aims. First, we examined whether the association between the economic recession caused by the 2007–2008 financial crisis and mental health disorders varied by sex. Second, we assessed whether the financial crisis had different effects on mental health depending on socioeconomic subgroups.

## Methods

### Sample

Data for this study were obtained from a nationwide, population-based survey—the “Longitudinal Survey of Middle-aged and Elderly Persons (LSMEP)”^25^. The LSMEP has been conducted by the Ministry of Health, Labour and Welfare in Japan since 2005. The original dataset is publicly available on the data depository of the Ministry of Health, Labour and Welfare with administrative permission.

A stratified two-stage sampling technique was used to select participants in the baseline survey in 2005: (i) 2515 census districts were randomly selected across Japan, and (ii) 40,877 people aged 50–59 years were randomly selected from the 2515 census districts. Of those selected, 34,240 responded to the baseline survey and were annually followed-up thereafter. No new participants have been added subsequently. The 2011 Great East Japan Earthquake in Japan had a major effect on people’s attitudes, behaviors, and health conditions. Therefore, we only analyzed data from 2005 to 2010 (i.e., 6 years of data from before the Great East Japan Earthquake) to minimize contamination. The response rates in the 2005–2010 surveys ranged from 83.8 to 97.3%.

The study protocol was approved by the Research Ethics Committee of the University of Tokyo (approval number: 19-167; approved on August 6, 2019) and the study performed in accordance with the principles of the Declaration of Helsinki. The use of the LSMEP data was approved by the Ministry of Health, Labour and Welfare based on Article 32 of the Statistics Act, rendering this study ineligible for review as per the Ethical Guidelines for Epidemiological Research of the Japanese government^26^. All participants provided informed consent and received a detailed written explanation of the study. Consent for participation was implied through the return of the completed questionnaire.

### Measures

The original LSMEP questionnaire was administered annually and included the following items.

#### Mental health

The Japanese version of the 6-item Kessler Psychological Distress Scale (K6) was used to assess mental health status. The K6 is a screening scale for psychological distress that can detect disorders classified in the Diagnostic and Statistical Manual of Mental Disorders, Fourth Edition (DSM-IV)^27,28^. The Japanese version of the K6 has been previously validated^27^. Respondents evaluated six items on a 5-point Likert scale and their responses were transformed into scores ranging from 0 to 4 points (score range: 0–24). A higher total score indicated poorer mental health. Using the optimal threshold that indicates moderate distress^29^, we regarded a score of ≥ 5 to denote mental health distress. Cronbach’s alpha was 0.88 in this study.

#### Economic recession

To capture the effects of the economic recession, we created a binary variable of the year that the survey was conducted; the two binary values were before and after the recession (i.e., “before 2007” and “after 2008”). Considering the financial crisis that brought about the economic recession, we chose 2008 as the mid-point. Japan’s economy shrank from 2008 to 2009 because of this crisis. Specifically, Japan’s GDP declined to negative figures in the second quarter of 2008, and sharply declined further in the fourth quarter^30^. As the LSMEP is conducted in November each year, we assumed that the 2008 survey showed the initial effects of the economic recession.

#### Socioeconomic variables

The SES determinants measured comprised educational level, employment status, and household income. We regarded SES as time constant. Educational level included the following four categories: “junior high school graduate,” “high school graduate,” “junior college graduate,” and “university/graduate school graduate.” Participants were also categorized according to the following four types of employment status: “full-time,” “part-time,” “self-employed,” and “unemployed.” Educational level and employment status in the 2005 survey were used in the analysis.

Finally, the average monthly household income between 2005 and 2007 was used as a household income variable. The monthly household income is the total sum of self-reported income of the respondent and their spouse. For the analysis, we divided the responses into four groups according to quartiles (from Q1 [lowest] to Q4 [highest]).

#### Covariates

We collected data on age, sex (“men” or “women”), marital status (“married” or “unmarried”), and experience of retirement/quitting a job (“Have you retired or quit your job in the past one year”; “yes” or “no”) as demographic variables. In addition, we included smoking (“yes” or “no”) and alcohol intake (“drinking every day” to “never/unable to drink”) as health behaviors, and self-rated health (“good” or “poor”) as health status, because previous research has established an association between these factors and mental health^31–33^. The response to the query on retirement/quitting a job was aggregated, and we generated the experience of retirement/quitting a job during the study period as a time-constant variable. The other covariates were used as time-varying variables (i.e., the variables measured in every survey were included in the model).

### Data analysis

A generalized estimating equation was applied with mental health status as the dependent variable. The analyses were stratified by sex because previous studies indicate that the effect of economic recessions on mental health differs by sex^34^. Among the 34,240 respondents, 33,815 who responded at least once before 2007 and after 2008 were included in the analysis under four models: Model 1 did not include any interaction terms; Model 2 included interaction terms between household income and year (after 2008); Model 3 included interaction terms between education level and year (after 2008); and Model 4 included interaction terms between employment status and year (after 2008).

To minimize the potential bias associated with item nonresponse, we conducted multiple imputation using the Markov Chain Monte Carlo method based on the missing-at-random assumption (10 datasets were generated)^35,36^. All the variables included in this study were used to impute the data. For the K6, we imputed scores on each scale item, and then calculated the total score in accordance with a previous study^37^.

The results are presented as odds ratios (ORs) with 95% confidence intervals (CIs). We analyzed the data using Stata/SE software version 14.2 (StataCorp LCC, College Station, TX, USA).

## Results

The baseline descriptive statistics are shown in Table 1. The average age across all participants was 54.7 years (standard deviation: 2.8 years), and 48.6% were men. Regarding education, 19.0%, 48.3%, 17.3%, and 15.4% had graduated from junior high school, high school, junior college, and university/graduate school, respectively. Regarding employment status, 36.2%, 22.0%, 22.4%, and 19.4% of participants were full-time, part-time, self-employed, and unemployed, respectively. The average monthly household income was 675 thousand yen (approximately equal to 4.5 thousand US dollars). The proportion of participants with the experience of retirement or quitting a job during the study period was 8.5% (data not shown in Table 1). This proportion was 12.0% in men and 5.1% in women.

**Table 1:** Characteristics of the study participants (Year 2005, N = 33,815, after imputation).

			All	Men	Women	Missing (All)
			N = 33,815	N = 16,416	N = 17,399	
Age (years)		Mean (SD)	54.7 (2.8)	54.7 (2.7)	54.7 (2.8)	0 (0.0%)
		Median	55.0	55.0	55.0	
Sex	Men	n (%)	16,416 (48.6%)			0 (0.0%)
Marital status	Married	n (%)	28,952 (85.6%)	14,251 (86.8%)	14,701 (84.5%)	851 (2.5%)
Education						
Junior high school graduate		n (%)	6412 (19.0%)	3150 (19.2%)	3262 (18.8%)	2809 (8.3%)
High school graduate		n (%)	16,333 (48.3%)	7558 (46.0%)	8774 (50.4%)	
Junior college graduate		n (%)	5852 (17.3%)	1692 (10.3%)	4160 (23.9%)	
University/graduate school graduate		n (%)	5218 (15.4%)	4016 (24.5%)	1203 (6.9%)	
Employment						
Full-time		n (%)	12,226 (36.2%)	9107 (55.5%)	3119 (17.9%)	0 (0.0%)
Part-time		n (%)	7446 (22.0%)	1419 (8.6%)	6027 (34.6%)	
Self-employed		n (%)	7571 (22.4%)	4804 (29.3%)	2767 (15.9%)	
Unemployed		n (%)	6572 (19.4%)	1086 (6.6%)	5486 (31.5%)	
Monthly household income (10,000 yen)		Mean (SD)	67.5 (81.0)	69.5 (86.9)	65.6 (75.0)	19,370 (57.3%)
		Median	50.0	51.0	48.0	
Smoking behavior	Smoking	n (%)	10,355 (30.6%)	8042 (49.0%)	2313 (13.3%)	393 (1.2%)
Alcohol intake						
Never/unable to drink		n (%)	16,545 (48.9%)	4446 (27.1%)	12,099 (69.5%)	366 (1.1%)
1–3 days a month		n (%)	2138 (6.3%)	905 (5.5%)	1233 (7.1%)	
1–2 days a week		n (%)	2271 (6.7%)	1146 (7.0%)	1125 (6.5%)	
3–4 days a week		n (%)	2299 (6.8%)	1450 (8.8%)	849 (4.9%)	
5–6 days a week		n (%)	2330 (6.9%)	1668 (10.2%)	662 (3.8%)	
Every day		n (%)	8232 (24.3%)	6800 (41.4%)	1432 (8.2%)	
Self-rated health	Poor	n (%)	6362 (18.8%)	3194 (19.5%)	3168 (18.2%)	277 (0.8%)
Mental health distress	K6 ≥ 5	n (%)	9489 (28.1%)	4400 (26.8%)	5088 (29.2%)	2310 (6.8%)

K6, 6-item Kessler Psychological Distress Scale; SD, standard deviation.

The proportion of people with mental health distress (a K6 score of ≥ 5) was 28.1%. Supplementary Table S1 compares demographic characteristics between participants with and without mental health distress. Those with mental health distress tended to have a lower level of education, were unemployed (or not working full-time), and had lower household incomes than participants without mental health distress.

Results of the generalized estimating equation are presented in Tables 2 and 3 for men and women, respectively. The Model 1 analysis indicated a deterioration in mental health status for both men and women after 2008. Men experienced a 1.11-fold increase in mental health distress after the economic crisis compared with before (OR [95% CI] = 1.11 [1.07–1.15]). Similarly, women underwent a 1.14-fold increase in mental health distress following the economic crisis (1.14 [1.10–1.18]).

**Table 2:** Multivariable analysis of associations between economic recession, socioeconomic status, and mental health distress in men.

		Model 1	Model 2	Model 3	Model 4
		OR (95% CI)	OR (95% CI)	OR (95% CI)	OR (95% CI)
After 2008 (Ref.: before 2007)		1.11 (1.07–1.15)	1.10 (1.06–1.15)	1.10 (1.06–1.15)	1.11 (1.07–1.15)
Age	(Older)	0.97 (0.96–0.98)	0.97 (0.96–0.98)	0.97 (0.96–0.98)	0.97 (0.96–0.98)
Married (Ref.: unmarried)		0.78 (0.72–0.84)	0.78 (0.72–0.84)	0.78 (0.72–0.84)	0.78 (0.72–0.84)
Experience of retirement/quitting a job during the study period (Ref.: no)		0.89 (0.83–0.96)	0.89 (0.83–0.96)	0.89 (0.83–0.96)	0.89 (0.83–0.96)
Smoking behavior (Ref.: non-smoking)		1.03 (0.98–1.08)	1.03 (0.98–1.08)	1.03 (0.98–1.08)	1.03 (0.98–1.08)
Alcohol intake	(More frequent)	0.99 (0.98–1.00)	0.99 (0.98–1.00)	0.99 (0.98–1.00)	0.99 (0.98–1.00)
Poor self-rated health (Ref.: good)		2.57 (2.48–2.68)	2.57 (2.48–2.68)	2.57 (2.48–2.67)	2.57 (2.48–2.68)
Education (Ref.: university/graduate school graduate)	Junior college graduate	1.08 (0.98–1.19)	1.08 (0.98–1.19)	1.07 (0.98–1.18)	1.08 (0.98–1.19)
	High school graduate	1.01 (0.95–1.08)	1.02 (0.95–1.08)	1.01 (0.95–1.08)	1.01 (0.95–1.08)
	Junior high school graduate	1.05 (0.97–1.14)	1.04 (0.96–1.13)	1.05 (0.97–1.14)	1.05 (0.97–1.14)
Employment (Ref.: full-time)	Part-time	1.17 (1.07–1.29)	1.17 (1.07–1.29)	1.17 (1.06–1.28)	1.17 (1.07–1.29)
	Self-employed	1.00 (0.98–1.11)	1.04 (0.98–1.11)	1.03 (0.97–1.10)	1.04 (0.98–1.11)
	Unemployed	1.56 (1.41–1.73)	1.56 (1.41–1.73)	1.57 (1.42–1.74)	1.56 (1.41–1.73)
Household income stratification (Ref.: Q4 [highest])	Q3	1.02 (0.90–1.16)	1.02 (0.90–1.16)	1.02 (0.90–1.16)	1.02 (0.90–1.16)
	Q2	1.11 (0.99–1.24)	1.11 (0.99–1.24)	1.11 (0.99–1.24)	1.11 (0.99–1.24)
	Q1 (lowest)	1.21 (1.07–1.38)	1.21 (1.07–1.38)	1.21 (1.07–1.38)	1.21 (1.07–1.38)
Interaction term					
Education (Ref.: university/graduate school graduate)	Junior college graduate		0.96 (0.86–1.06)		
	High school graduate		0.96 (0.90–1.02)		
	Junior high school graduate		1.03 (0.95–1.11)		
Employment (Ref.: full-time)	Part-time			1.04 (0.95–1.15)	
	Self-employed			1.08 (1.02–1.14)	
	Unemployed			0.95 (0.85–1.05)	
Household income stratification (Ref.: Q4 [highest])	Q3				0.99 (0.89–1.09)
	Q2				1.02 (0.94–1.10)
	Q1 (lowest)				1.00 (0.91–1.09)
Constant term		0.38 (0.37–0.40)	0.38 (0.37–0.40)	0.38 (0.37–0.40)	0.38 (0.37–0.40)

CI, confidence interval; OR, odds ratio.

**Table 3:** Multivariable analysis of associations between economic recession, socioeconomic status, and mental health distress in women.

		Model 1	Model 2	Model 3	Model 4
		OR (95% CI)	OR (95% CI)	OR (95% CI)	OR (95% CI)
After 2008 (Ref.: before 2007)		1.14 (1.10–1.18)	1.15 (1.11–1.19)	1.13 (1.09–1.17)	1.14 (1.10–1.18)
Age	(Older)	0.97 (0.97–0.98)	0.97 (0.97–0.98)	0.97 (0.97–0.98)	0.97 (0.97–0.98)
Married (Ref.: unmarried)		0.83 (0.79–0.88)	0.83 (0.79–0.88)	0.83 (0.79–0.88)	0.83 (0.79–0.88)
Experience of retirement/quitting a job during the study period (Ref.: no)		0.94 (0.83–1.06)	0.94 (0.83–1.06)	0.94 (0.83–1.06)	0.94 (0.83–1.06)
Smoking behavior (Ref.: non-smoking)		1.19 (1.12–1.26)	1.19 (1.12–1.26)	1.19 (1.12–1.26)	1.19 (1.12–1.26)
Alcohol intake	(More frequent)	1.00 (0.99–1.02)	1.00 (0.99–1.02)	1.00 (0.99–1.02)	1.00 (0.99–1.02)
Poor self-rated health (Ref.: good)		2.56 (2.47–2.66)	2.56 (2.47–2.66)	2.56 (2.47–2.66)	2.56 (2.47–2.66)
Education (Ref.: university/graduate school graduate)	Junior college graduate	1.00 (0.91–1.09)	1.00 (0.91–1.10)	1.00 (0.91–1.09)	1.00 (0.91–1.09)
	High school graduate	0.95 (0.87–1.04)	0.96 (0.88–1.05)	0.95 (0.87–1.04)	0.95 (0.87–1.04)
	Junior high school graduate	1.02 (0.93–1.13)	1.04 (0.94–1.14)	1.02 (0.93–1.13)	1.03 (0.93–1.13)
Employment (Ref.: full-time)	Part-time	1.00 (0.93–1.07)	1.00 (0.93–1.07)	0.99 (0.92–1.07)	1.00 (0.93–1.07)
	Self-employed	1.04 (0.96–1.14)	1.04 (0.96–1.14)	1.04 (0.96–1.14)	1.04 (0.96–1.14)
	Unemployed	1.13 (1.04–1.22)	1.13 (1.05–1.22)	1.12 (1.04–1.21)	1.13 (1.04–1.22)
Household income stratification (Ref.: Q4 [highest])	Q3	1.01 (0.91–1.14)	1.02 (0.91–1.14)	1.01 (0.91–1.14)	1.02 (0.90–1.14)
	Q2	1.05 (0.95–1.16)	1.05 (0.95–1.16)	1.05 (0.95–1.16)	1.05 (0.94–1.16)
	Q1 (lowest)	1.12 (1.00–1.26)	1.13 (1.00–1.27)	1.12 (1.00–1.26)	1.13 (1.00–1.27)
Interaction term					
Education (Ref.: university/graduate school graduate)	Junior college graduate		0.93 (0.85–1.02)		
	High school graduate		0.92 (0.84–1.01)		
	Junior high school graduate		0.89 (0.80–0.98)		
Employment (Ref.: full-time)	Part-time			1.04 (0.97–1.11)	
	Self-employed			0.99 (0.92–1.08)	
	Unemployed			1.04 (0.97–1.11)	
Household income stratification (Ref.: Q4 [highest])	Q3				1.00 (0.92–1.08)
	Q2				1.00 (0.92–1.08)
	Q1 (lowest)				0.99 (0.91–1.08)
Constant term		0.43 (0.42–0.45)	0.43 (0.42–0.45)	0.43 (0.42–0.45)	0.43 (0.42–0.45)

CI, confidence interval; OR, odds ratio.

**Fig. 1:**
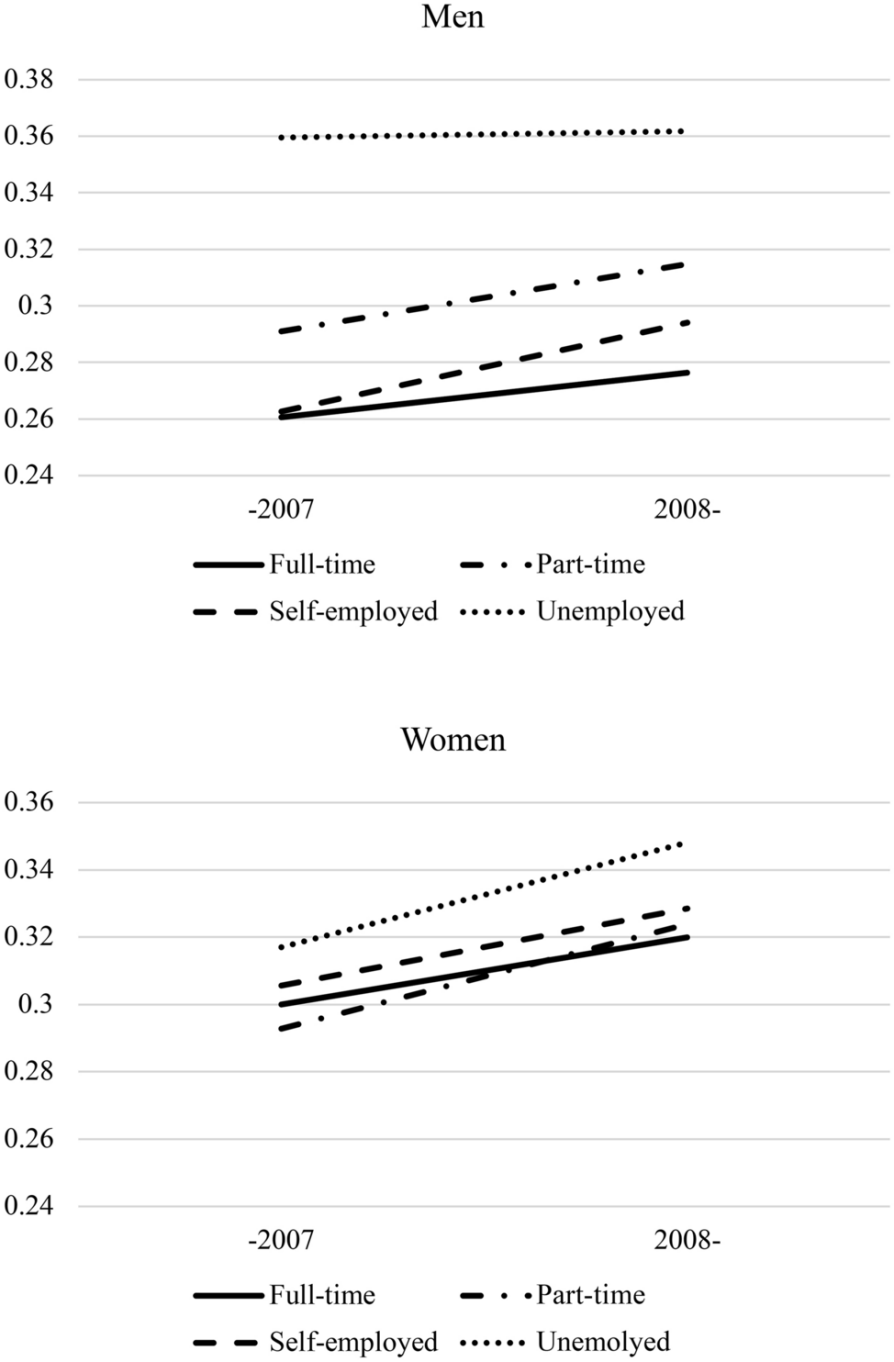
Economic recession, employment status, and mental health distress (men and women).

**Fig. 2:**
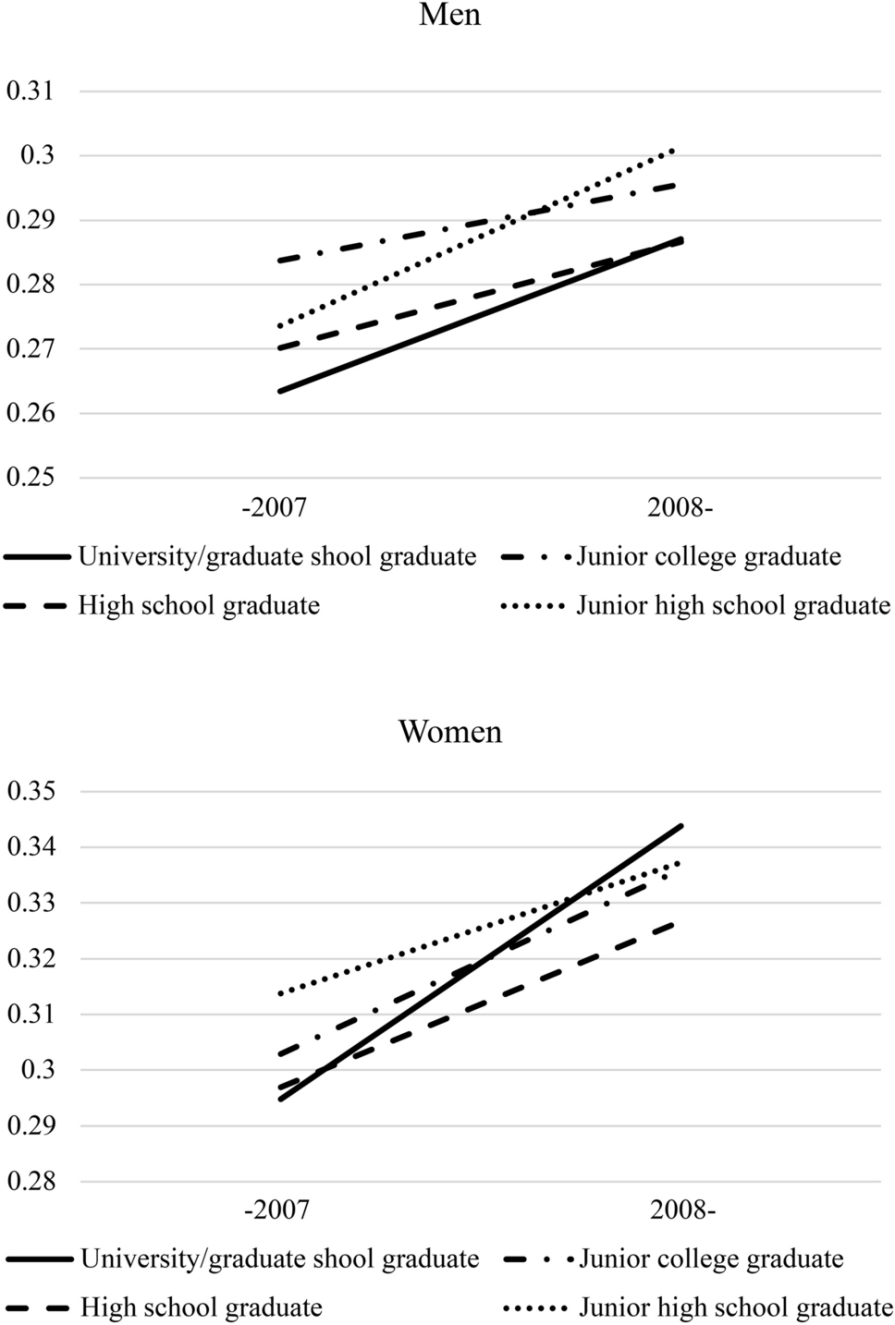
Economic recession, educational background, and mental health distress (men and women).

Using the interactions between the year (i.e., after 2008) and socioeconomic variables, Figs. 1 and 2 show the prevalence of mental health distress by subgroup at two time points—before the financial crisis (2005–2007) and after the financial crisis (2008–2010). Men who were self-employed tended to have worse mental health after the recession (1.08 [1.02–1.14]; Model 3 of Table 2 and Fig. 1). In women, mental health deterioration after the recession was lower among those who graduated from junior high school compared with those who graduated from university or graduate school (0.89 [0.80–0.98]; Model 2 of Table 3 and Fig. 2). In other words, the mental health of highly educated women deteriorated more severely after the recession. However, the interaction between the year variable and average household income was not statistically significant for men or women.

## Discussion

Controlling for potential confounders, this study showed that mental health among a large sample of Japanese middle-aged adults deteriorated after the 2008 economic recession. Self-employed men were found to have experienced more severe mental health deterioration than those with full-time jobs. Highly educated women (university/graduate school graduates) experienced greater mental health deterioration than their less-educated counterparts.

This study confirmed that the experience of the economic recession caused by the financial crisis of 2007–2008 was associated with increased mental health distress among the Japanese population. This trend is similar to findings in previous studies from other countries^2,5,10,13,14,17–19,22–24,38,39^. Mechanisms suggested in previous research include financial hardships such as increasing debt, lower income, and job insecurity^8^.

The effect of SES (educational background, employment status, and household income stratification) on the association between the recession and mental health was observed as two disproportionate effects of the recession. First, no change was observed in the mental health of participants who were unemployed before and after the recession; however, the mental health of self-employed participants, particularly men, was severely affected. It is reasonable to assume that participants who withdrew from the labor market prior to the recession were not affected by the changing economic conditions, whereas those who were vulnerable in the economic market were highly affected. A previous study reported that self-employed people were less resilient to income losses and those in households with below-adequate incomes reported greater concerns about social cohesion^40^. Moreover, in Japan, self-employed men still tend to be the breadwinners in their households^41^. Therefore, economic recession might have a particularly negative effect on the mental health of self-employed men.

Second, the mental health of highly educated women deteriorated more severely after the recession. Previous studies have reported that a lower level of education is associated with a lack of control and resilience toward mental health problems, and that the tendency to seek appropriate mental health support services differs according to educational levels^42,43^. Furthermore, a study found that people with low education had poorer mental health literacy than highly educated people^44^. However, our research found contradictory results. During the study period, the labor force in Japan was changing owing to the increasing participation and assimilation of women, who experience relatively stressful labor market conditions^45,46^. Women also experience stress from balancing work and home responsibilities, particularly highly educated women, who have chosen unconventional careers and roles in their organizations. A previous study reported that more educated women experienced greater work–family conflict than their less-educated counterparts^47^. This may explain why highly educated women in our study experienced a severe decline in mental health after the recession. A better understanding of the different effects of socioeconomic background may be obtained by focusing on minority groups, who may have different education and work backgrounds, as well as more involvement of family dynamics.

This research had several limitations. First, the effectiveness of the year as a variable to assess the effects of economic recession cannot be confirmed. However, this study confirmed the findings of prior research that the recession had a negative effect on mental health. Second, we did not include other related factors such as policy and environmental changes, and macroeconomic rates; future research should include these factors to obtain a more holistic understanding of the effects of economic recession. Third, we focused only on the association between time (i.e., the survey year) and mental health in the Japanese population in the context of the 2007–2008 financial crisis and resulting recession. Therefore, the causal relationship between economic crises and mental health has not been proven.

## Conclusion

This study analyzed the effects of the recession following the 2007–2008 financial crisis on mental health by sex and SES. The findings have implications for managing economic recessions in general, and highlight socioeconomically vulnerable groups in terms of employment status and educational background. Our study underscores the importance of monitoring the effects of economic recessions and providing tailored and timely support for these groups so as to minimize the negative effects of economic recessions.

## Supplementary Information

Supplementary Information.

## Data Availability

The original dataset of the LSMEP is publicly available on the data depository of the Ministry of Health, Labour and Welfare with administrative permission (URL: https://www.e-stat.go.jp/en/statistics/00,450,045; contact office: Household Statistics Office).
