# Supplementary material for: Economic recession and mental health distress among Japanese people in middle age
Source: Sci Rep. 2025 Apr 16;15:13190. doi: 10.1038/s41598-025-85198-6 (PMC12003673; doi:10.1038/s41598-025-85198-6)
Supplement: Supplementary file 1 — Supplementary Information. [file 41598_2025_85198_MOESM1_ESM.docx]

| **Supplementary Table S1.** Baseline characteristics of the study participants by mental health status (Year 2005, N = 33,815, after imputation) | | | | | | |
| --- | --- | --- | --- | --- | --- | --- |
|  | | | | Poor mental health status  (K6 score ≥5) | Good mental health status  (K6 score <5) | p-value |
|  |  |  |  | N = 9,489 | N = 24,326 |  |
| Age (years) | |  | Mean (SD) | 54.5 (2.7) | 54.7 (2.7) | <0.001 |
| Sex | | Men | n (%) | 4,400 (46.4%) | 12,016 (49.4%) | <0.001 |
| Marital status | | Married | n (%) | 7,795 (82.2%) | 21,157 (87.0%) | <0.001 |
| Education | |  |  |  |  |  |
|  | Junior high school graduate |  | n (%) | 1,937 (20.4%) | 4,475 (18.4%) | <0.001 |
|  | High school graduate |  | n (%) | 4,507 (47.5%) | 11,826 (48.6%) |  |
|  | Junior college graduate |  | n (%) | 1,729 (18.2%) | 4,123 (16.9%) |  |
|  | University/graduate school graduate |  | n (%) | 1,316 (13.9%) | 3,902 (16.0%) |  |
| Employment | |  |  |  |  |  |
|  | Full-time |  | n (%) | 3,171 (33.4%) | 9,055 (37.2%) | <0.001 |
|  | Part-time |  | n (%) | 2,085 (22.0%) | 5,361 (22.0%) |  |
|  | Self-employed |  | n (%) | 1,995 (21.0%) | 5,576 (22.9%) |  |
|  | Unemployed |  | n (%) | 2,237 (23.6%) | 4,335 (17.8%) |  |
| Monthly household income (10,000 yen) | |  | Mean (SD) | 63.7 (51.6) | 69.0 (58.1) | <0.001 |
| K6, 6-item Kessler Psychological Distress Scale; SD, standard deviation. | | | | | | |
